# Treatment of Disseminated Mycobacterial Infection with High-Dose IFN-***γ*** in a Patient with IL-12R*β*1 Deficiency

**DOI:** 10.1155/2011/691956

**Published:** 2010-12-22

**Authors:** Abdullah A. Alangari, Fahad Al-Zamil, Abdulrahman Al-Mazrou, Saleh Al-Muhsen, Stéphanie Boisson-Dupuis, Sitalbanat Awadallah, Abdelmageed Kambal, Jean-Laurent Casanova

**Affiliations:** ^1^Department of Pediatrics, College of Medicine, King Saud University, P.O. Box 2925, Riyadh 11461, Saudi Arabia; ^2^Laboratory of Human Genetics of Infectious Diseases, Necker Branch, Inserm, U550, 75654 Paris, France; ^3^Laboratory of Human Genetics of Infectious Diseases, Rockefeller Branch, The Rockefeller University, St. Giles, NY 10065, USA; ^4^Department of Physiology, College of Medicine, King Saud University, Riyadh 11461, Saudi Arabia; ^5^Department of Pathology, College of Medicine, King Saud University, Riyadh 11461, Saudi Arabia

## Abstract

IFN-*γ* has been used in the treatment of IL-12R*β*1 deficiency patients with disseminated BCG infection (BCGosis), but the optimal dose to reach efficacy is not clear. We used IFN-*γ* in the treatment of a 2.7-year-old patient with IL-12R*β*1 deficiency and refractory BCG-osis. IFN*γ* was started at a dose of 50 *μ*g/m^2^ 3 times per week. The dose was upgraded to 100 mcg/m^2^ after 3 months, then to 200 mcg/m^2^ 6 months afterwards. Serum mycobactericidal activity and lymphocytes number and function were evaluated throughout the study. There was no clinical response to IFN-*γ* with 50 or 100 *μ*g/m^2^ doses. However, there was some response to the 200 *μ*g/m^2^ dose with no additional adverse effects. The serum mycobactericidal activity was not significantly different during the whole treatment period. Lymphocytes proliferation in response to PHA was significantly higher after 3 months of using the highest dose as compared to the lowest dose. The tuberculin skin test reaction remained persistently negative. We conclude that in a patient with IL-12R*β*1 deficiency, IFN-*γ* at a dose of 200 *μ*g/m^2^, but not at lower dosages, was found to have a noticeable clinical effect with no additional adverse effects.

## 1. Introduction

Investigation of a human syndrome known as Mendelian susceptibility to mycobacterial diseases (MSMD) (OMIM 209950) has, in the past 15 years, led to the identification of a series of genetic defects in the IL-12/IFN-*γ* axis. These include defects in three autosomal genes controlling the response to IFN-*γ*: *IFNGR1*, encoding the ligand binding, first chain of the IFN-*γ* receptor; *IFNGR2*, encoding the signaling, second chain of the IFN-*γ* receptor; *STAT1*, encoding the signal transducer and activator of transcription 1 downstream from IFN-*γ* receptor. They also include defects in two other autosomal genes controlling the production of IFN-*γ*: *IL12B*, encoding IL-12p40 shared by IL-12 and IL-23; *IL12RB1*, encoding the first chain of the IL-12 and IL-23 receptor (IL-12R*β*1). In addition, there is one X-linked gene that encodes nuclear factor-kB essential modulator (*NEMO*) [1–3].

IL-12R*β*1 defect was first described in 1998 [4] and is the most common among the known genetic disorders that predispose to mycobacterial infections. The spectrum of infections reported in these patients is, however, surprisingly narrow. These patients display selective susceptibility to weakly virulent *Mycobacteria*, such as environmental mycobacteria (EM) and live bacille Calmette-Guérin (BCG) vaccine (an attenuated substrain of *Mycobacterium bovis*), and *Salmonella*. They showed almost no increased susceptibility to other pathogens including other bacteria and ubiquitously distributed viruses and fungi [5]. Evidence suggests that the immune system is redundant in its response to many intracellular microorganisms. For other organisms like varicella zoster virus, Th1 activation and IFN-*γ* production was found to be stimulated by IFN-*α* not IL-12 [6]. 

In a very recent report, that included the largest cohort of patients with IL-12R*β*1 deficiency (141 patients) [7], the mean age of onset of the first infection was 2.4 years. The severity of the disease varied significantly from subjects who were asymptomatic until adulthood to patients who died in early childhood from complications of the disease. The mortality rate was 32% among symptomatic subjects and the mean age of death was 7.5 years, mostly secondary to BCGosis or EM disease.

In patients with IL-12R*β*1 deficiency who do not respond to prolonged therapy with multiple anti-mycobacterial drugs, the mycobacteria frequently develop resistance to some of these medications. The management of those patients subsequently becomes very difficult and frequently they will not recover using antimycobacterials alone. Since the most important reason of those patients' failure to control mycobacterial infections is the inability of their T cells and NK cells to produce IFN-*γ* in response to IL-12 stimulation, IFN-*γ* is an attractive candidate therapy that can be tried in those patients [8]. However, the optimal dose and duration of using IFN-*γ* are not clear.

## 2. Subject and Methods

### 2.1. Study Patient

Our patient was referred to us at 6 months of age with left axillary lymphadenitis that grew the vaccine strain of *Mycobacterium bovis *(BCG), a routine vaccine given in day one of life in Saudi Arabia. There was no granuloma formation on the lymph node biopsy. She was otherwise well apart from intermittent fever and night sweats. Her parents were not consanguineous but from the same tribe. She had a 5-years-old brother who had left axillary BCGitis in infancy that resolved promptly after treatment with isoniazid alone for 6 months. She also had a 7-years-old sister who has been completely healthy. She did not respond to isoniazide and rifampicin. Sensitivity results then showed that the organism was resistant to both, so she was started on ethambutol 300 mg OD, cycloserine 250 mg OD, and moxifloxacin 200 mg OD for which the organism was sensitive. Meanwhile, immunological investigations revealed that our patient's lymphocytes have no IFN-*γ* production in response to IL-12+BCG as compared to controls (those investigations were performed in Dr. Casanova's lab in Paris. Her parents were travel controls). Her brother had a similar cellular phenotype (i.e., no IFN-*γ* production after BCG and BCG+IL-12 stimulation in whole-blood assay) (Table 1). In addition, no cell surface-expressed IL12RB1 could be detected from their PHA-T cell blast (data not shown). Genetic testing confirmed that she and her brother have homozygous 1336delC mutation in the *IL12RB1* gene, leading to complete IL12R*β*1 deficiency. Her sister was functionally and genetically normal. To our knowledge this is the only family with this specific type of mutation to be reported [7].

### 2.2. Study Plan

After parental consent, baseline and followup investigations were obtained including CBC, ESR, liver enzymes, lymphocytes subsets, and tuberculin skin test (TST) every 3 months. Immunoglobulin levels, serum mycobactericidal activity, and lymphocytes proliferation in vitro were performed every 6 months. 

The patient was seen in the clinic every 6 weeks. IFN-*γ*-1b (Imukin, Boehringer Ingelheim) was started at a dose of 50 *μ*g/m^2^ subcutaneously 3 times/week. If there is no significant response within 3 months the dose will be doubled to 100 mcg/m^2^, at the same frequency.

### 2.3. Cellular Studies

Blood samples were taken from the patient before starting treatment with IFN*γ* (S1), 6 months (S2) and 12 months (S3) after starting treatment. PBMCs were isolated from whole blood by density centrifugation (Lymphoprep, Nycomed, Oslo, Norway) and assayed for in vitro proliferative responses by the thymidine incorporation method against phytohemagglutinin (PHA) (Sigma, St. Louis, Mo.) and IL2 (R&D System, Abington, UK). 

The proliferation results are expressed as mean count per minute of triplicate cultures for the antigen concentration giving maximum response minus the mean count-per-minute values for 12 wells without antigen (medium only).

### 2.4. Serum Mycobactericidal Assay

Two specimens of the patient's serum were collected at 6 months and 12 months of the study period, 2 hours after anti-mycobacterial drug administration, to determine the serum inhibitory and bactericidal titers against the patient's mycobacterial isolate as well as mycobacterium bovis BCG vaccine strain (Statens Serum Institute, Copenhagen S, Denmark) and mycobacterium tuberculosis H37RV reference strain (ATCC, Atlanta, GA, USA), as previously described [9].

## 3. Results

**Figure 1:**
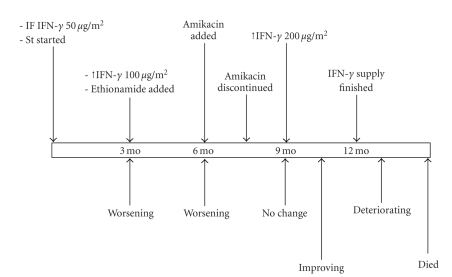
Time line of the patient's course. Above are the changes in medications and below are the changes in clinical condition from the beginning of IFN-*γ* introductions till the patients' death 15 months later.

Our patient was started on IFN-*γ* at a dose of 50 *μ*g/m^2^ three times a week in addition to her anti-mycobacterial medications as above (Figure 1). Three months later, the patient's condition was slightly worse, so ethionamide 250 mg OD was added to her drug regimen and IFN-*γ* dose was upgraded to 100 *μ*g/m^2^ three times a week. Six months from the beginning of the study her clinical condition continued to worsen and she developed a left chest wall abscess. At that time amikacin was added to her drug regimen for 6 weeks only. She was continued on the same dose of IFN-*γ*. Nine months from the beginning of the study there was no clinical improvement. She was on four anti-mycobacterial medications as mentioned above. At this point the dose was upgraded to 200 *μ*g/m^2^. One month later the enlarged lymph nodes and the chest wall abscess started to discharge pus that was positive for acid-fast bacilli on ZN stain and the culture grew *Mycobacterium bovis*. The patient felt better and the abscesses and the discharging lymph nodes healed. One month later she developed right pleural effusion, but remained clinically stable. Cultures from the effusion were negative. After 12 months from the beginning of the study we ran out of IFN-*γ* and the patient continued on her usual anti-mycobacterial medications. She then started to deteriorate gradually. Three months later she developed massive pneumonia and died. The only adverse effect from IFN-*γ* therapy that was noted throughout the study at the different doses was fever (up to 39°C) and lethargy up to 8 hours from giving the injection. Her CBC, liver enzymes, lymphocyte subsets, and immunoglobulin levels were not significantly changed and her ESR fluctuated between 80–107 mm/hr throughout the study period.

**Figure 2:**
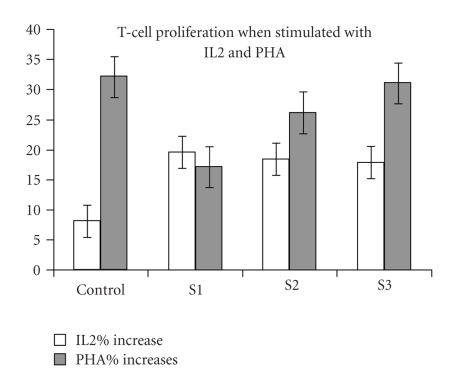
The percent increase in T cell proliferation stimulated with PHA and IL2 in control and the three samples from the patient: (S1) before treatment, (S2) 6 months after treatment (S3) 12 months after treatment. The percent increase in Sample, (S3) stimulation with PHA was significantly higher than the response before treatment (S1) (*P* = .02), while no significant difference with IL2.

The spectrum of proliferative responses of T cell following stimulation with PHA and IL-2 is shown in Figure 2. The percent increase of T cell proliferation in response to PHA (1 : 10 dilution) rose from 17% at baseline to 26% at 6 months of the study, reaching 32% at 12 months (*P* = .02) after increasing the dose of IFN-*γ* to 200 *μ*g/m^2^. There was, however, no change after IL-2 stimulation, where percent increase of T cell proliferation was 20%, 18%, and 17% at baseline, 6 months, and 12 months, respectively.

There was no significant difference between the serum mycobactericidal activity at 6 months and at 12 months.

## 4. Discussion

Our patient had a severe phenotype of IL-12R*β*1 deficiency. In addition, the organism was resistant to anti-BCG antibiotics. Therefore, it was more stringent to try IFN-*γ* as a therapeutic agent. IFN-*γ* was tried initially at a dose of 50 *μ*g/m^2^ 3 times a week based on the experience of using it in other conditions like chronic granulomatous disease (CGD) [10]. However, it was not surprising that it did not work at this concentration with our patient since, unlike patients with CGD, she is not able to produce IFN-*γ* and the pathophysiology of the disease is completely different. The clinical effect noted after using the high-dose IFN-*γ* (200 *μ*g/m^2^) may be attributed to this medication. This could be supported by the deterioration in the patient's condition after cessation of IFN-*γ*. Since there was no difference in the mycobactericidal activity between 6 months and 12 months of the study period, the changes in the patient's clinical condition are unlikely to be due to the anti-mycobacterial medications. 

The increase in the lymphocyte proliferation in response to PHA at 12 months as compared to 0 and 6 months may be secondary to indirect stimulation of the patient's lymphocytes with the high-dose IFN-*γ*. However, the persistently negative tuberculin skin test suggests that IFN-*γ* therapy did not improve specific DTH to mycobacterial PPD antigens. Since there were no additional adverse effects with the 200 *μ*g/m^2^ dose of IFN-*γ*, our experience with this patient may encourage physicians to start directly with this dose especially when there is no good response to maximum anti-mycobacterial therapy after few months of treatment.

Achieving clinical resolution is very important in the management of patients with IL-12R*β*1 deficiency and BCGosis. Patients with BCG disease rarely have recurrence or develop EM disease, if they responded to multidrug antibiotic treatment [7]. On the other hand, patients frequently develop recurrence of salmonellosis, sometimes involving the same serotype. Interestingly, more than a quarter of the genetically affected siblings remain asymptomatic. This strongly suggests that the IL-12 pathway is redundant for the primary immune response against *Mycobacteria* and *Salmonella* in a substantial proportion of patients. 

IFN-*γ* was tried in some patients with IL-12R*β*1 deficiency and BCGosis. There are only two reports of three patients where IFN-*γ* was tried. The first report includes 2 patients with BCGosis treated with IFN-*γ* for 18 months in addition to antimycobacterials [11]. One patient survived and the other died. The patient who responded had maintained a normal number of Th cells during treatment and had good proliferative response to mycobacterial antigens in vitro compared to the other patient. The second report included one patient who had BCGosis for 13 years and failed multiple first- and second-line antimycobacterials. She was having chronic diarrhea and her serum showed no in vitro bactericidal nor bacteriostatic activity against BCG. She fully recovered when IFN-*γ* was used for one year plus the addition of intravenous anti-mycobacterial treatment to her regimen [9]. Two other patients with similar presentation were treated similarly by the same group and did well (direct communication with Dr. Rosenzweig).

In many countries around the world BCG vaccine is given at birth or few months afterword. In Saudi Arabia, BCG vaccine is given in day 1 of life. This was mainly because of high incidence (243/100,000 in 1978) and problems with compliance with vaccination schedule. Subsequently, the incidence dropped to 90/100,000 in 1990 and now 11/1000,000 (Ministry of Health statistics). Recent studies showed that giving BCG vaccine at birth induces significant mycobacterial immune response as early as 2 months of life. When this response was tested at about 9 months of age it was found to be maintained and comparable to infants who received the vaccine at 2 or 4.5 months of life [12, 13]. 

In conclusion, in one patient with IL-12R*β*1 deficiency, IFN-*γ* at a dose of 200 *μ*g/m^2^, but not at lower dosages, was found to have a positive clinical effect with no additional adverse effects. This medication holds promise in the management of such patients especially if used early in the course of disease. Multicenter studies are needed to establish the effectiveness, dose, and duration of IFN-*γ* treatment on a large number of patients.

##  Conflict of Interest

The authors declare no conflict of interest.

## Figures and Tables

**Table 1:** In vitro Lymphocytes' IFN-*γ* production in different conditions.

Stimulant	IFN-*γ* (pg/ml)					
	Control	Father	Mother	Sister	Brother	Patient
Medium	0	62	62	0	0	0
BCG	738	202	68	47	35	0
BCG+IL12	44461	6000	4233	988	13	0
